# Validation of the plasmid study to relate DNA damaging effects of radionuclides to those from external beam radiotherapy

**DOI:** 10.1016/j.nucmedbio.2021.06.004

**Published:** 2021-06-15

**Authors:** Elise Verger, Jordan Cheng, Vittorio de Santis, Madeleine Iafrate, Jessica A. Jackson, Cinzia Imberti, Gilbert O. Fruhwirth, Philip J. Blower, Michelle T. Ma, Daniel R. Burnham, Samantha Y.A. Terry

**Affiliations:** a School of Biomedical Engineering and Imaging Sciences, King’s College London, St. Thomas’ Hospital, London SE1 7EH, United Kingdom; b Comprehensive Cancer Centre, School of Cancer and Pharmaceutical Sciences, King’s College London, Guy’s Hospital Campus, London SE1 1UL, United Kingdom; c Single Molecule Imaging of Genome Duplication and Maintenance Laboratory, The Francis Crick Institute, London, United Kingdom

**Keywords:** Radionuclides, External beam radiation, Radiobiology, Biological effects, Auger electrons

## Abstract

**Introduction:**

The biological consequences of absorbed radiation doses are ill-defined for radiopharmaceuticals, unlike for external beam radiotherapy (EBRT). A reliable assay that assesses the biological consequences of any radionuclide is much needed. Here, we evaluated the cell-free plasmid DNA assay to determine the relative biological effects of radionuclides such as Auger electron-emitting [^67^Ga]GaCl_3_ or [^111^In]InCl_3_ compared to EBRT.

**Methods:**

Supercoiled pBR322 plasmid DNA (1.25 or 5 ng/μL) was incubated with 0.5 or 1 MBq [^67^Ga]GaCl_3_ or [^111^In]InCl_3_ for up to 73 h or was exposed to EBRT(^137^Cs; 5 Gy/min; 0-40 Gy). The induction of relaxed and linear plasmid DNA, representing single and double strand breaks, respectively, was assessed by gel electrophoresis. Chelated forms of ^67^Ga were also investigated using DOTA and THP. Topological conversion rates for supercoiled-to-relaxed $\left(k_{sr}^{x}\right)$ or relaxed-to-linear $\left(k_{rl}^{\chi }\right)$ DNA were obtained by fitting a kinetic model.

**Results:**

DNA damage increased both with EBRT dose and incubation time for [^67^Ga]GaCl_3_ and [^111^In]InCl_3_. Damage caused by [^67^Ga]GaCl_3_ decreased when chelated. [^67^Ga]GaCl_3_ proved more damaging than [^111^In]InCl_3_; 1.25 ng/μL DNA incubated with 0.5 MBq [^67^Ga]GaCl_3_ for 2 h led to a 70% decrease of intact plasmid DNA as opposed to only a 19% decrease for [^111^In]InCl_3_. For both EBRT and radionuclides, conversion rates were slower for 5 ng/μL than 1.25 ng/μL plasmid DNA. DNA damage caused by 1 Gy EBRT was the equivalent to damage caused by 0.5 MBq unchelated [^67^Ga]GaCl_3_ and [^111^In]InCl_3_ after 2.05 ± 0.36 and 9.3 ± 0.77 h of incubation, respectively.

**Conclusions:**

This work has highlighted the power of the plasmid DNA assay for a rapid determination of the relative biological effects of radionuclides compared to external beam radiotherapy. It is envisaged this approach will enable the systematic assessment of imaging and therapeutic radionuclides, including Auger electron-emitters, to further inform radiopharmaceutical design and application.

## 1 Introduction

The use of external beam radiotherapy (EBRT) in the management and treatment of cancer has been a longstanding therapeutic strategy for almost 50% of all cancer patients [1]. However, a major concern with EBRT is the lack of tumour-specific targeting, resulting the irradiation of healthy tissues. This is especially problematic if the tumour is located near radiosensitive and healthy tissues or organs, such as the kidneys or bone marrow [2,3]. Consequently, not all cancerous lesions can be treated using EBRT [3]. Molecular radionuclide therapy (MRT), where injected radioactive compounds localise specifically to areas of interest, can provide away of targeting disseminated disease and potentially having very few side effects [3]. There are several successful clinical examples of MRT emitting short-range alpha or longer-range beta particles. For instance, the alpha particle-emitter, [^223^Ra]RaCl_2_, has been shown to successfully relieve pain from prostate cancer spread to the bone and increase overall survival by several months in patients with castration-resistant disease prostate cancer [4]. Also, beta emitting radiopharmaceutical [^131^I]-NaI can be used in its ionic form to target and ablate thyroid and thyroid cancer cells [5], and ^131^I in other forms can be used to treat other cancer types (e.g. [^131^I]-mIBG, which targets neuroendocrine tumours) [6]. Gastro-intestinal neuroendocrine tumours can also be treated with beta particle-emitting [^177^Lu] Lu-DOTATATE [7].

In nuclear medicine, patient dosimetry calculations are mainly performed to ensure radiation dose limits to healthy tissues are not breached during radionuclide imaging, such as positron emission tomography (PET) and single photon emission computed tomography (SPECT). Dosimetry is also used to calculate required injected activities of radiopharmaceuticals for successful therapeutic efficacy whilst adhering to radiation dose limits in organs such as the kidneys and bone marrow [8]. The radiation absorbed dose, quantified in Gray (Gy), is a measure of energy deposited in a medium by ionising radiation per unit mass. Notably, it cannot predict biological effects of radionuclides in tissues; for example, 1 Gy of EBRT may differently impede tumour growth compared to 1 Gy of targeted alpha particle radiotherapy.

A general consensus concerning the relative biological effectiveness (RBE) of MRT (encompassing alpha and beta particle-emitters) as compared to EBRT was reached in the late 1990’s. It was decided that if EBRT had a radiation weighting factor of 1, then, for example, alpha particle-emitting MRT should have a radiation weighting factor of 20 [9,10]. However, MRT encompasses many different radionuclides, with multiple types of alpha and beta particle-emitters, each with different emissions and energies. As such, the radiation weighting factor used to place biological effects from radiation exposure on a common scale is a nominal one that is unlikely to be the same for every alpha and beta particle-emitter within the broad classification of “MRT”. For example, 2 Gy of absorbed radiation dose is accepted as the dose limit to the bone marrow for MRT, as determined from studies using beta particle-emitter [^131^I]-NaI. However, dose limits between isotopes are not comparable and by taking [^131^I]-NaI as the guide might be setting it too low [11]. This highlights the need to reconsider dose limits for each individual radionuclide. Equally, as radiopharmaceuticals are targeted to different tissues and show non-uniform distribution within these tissues, thus affecting certain organs more than others, similar recalculations should be carried out for each and every radiopharmaceutical.

MRT has mostly focussed on radionuclides emitting alpha or beta particles. Auger electron MRT has recently come to the fore due to its therapeutic potential for micrometastases or single circulating tumour cells in cancer [12]. The range of Auger electrons does not usually extend past 1 μm, so their maximum therapeutic efficacy will be achieved when the Auger electron-emitters are targeted close to organelles of cellular importance; a prime example is the cell’s nucleus [13–18]. Our group has previously shown how therapeutic efficacy of Auger electron-emitters depends on their proximity to cellular DNA using a cell-free plasmid DNA assay. This assay showed that the Auger electron-emitters ^67^Ga and ^111^In caused less damage when chelated with EDTA or DTPA, respectively, i.e. when further away from the DNA molecules [19]. It was suggested that chelating radionuclides prevented their positively charged ions from binding (coordinating or ion-pairing) to the negatively charged DNA and/or created an electrostatic repulsion between the negatively charged chelates and DNA’s phosphate backbone. Historically, the majority of work relating to Auger electron-emitters has focussed on [^125^I]I [14,20–25], although [^111^In]In-Octreotide and [^111^In]In-EGF have come closest to clinical translation [26,27]. More recently, radiopharmaceuticals such as [^123^I]I-MAPi and [^125^I]I-DCIBzL intended to treat brain tumours and prostate cancer, respectively, are proving exciting avenues to explore the use of Auger electron-emitters in the clinic [28–30].

Redefining dose limits for radionuclides emitting Auger electrons is intrinsically linked to recent work, which accurately recalculates their S values by including the contribution of these low energy electrons [31,32]. In internal dosimetry, S values describe the mean absorbed dose to target regions per radioactive decay in the source region and as such, they are key in calculating total radiation absorbed dose. Recently, it was demonstrated that despite Auger electrons potentially delivering a higher radiation dose to dose-limiting organs such as the kidneys, this did not necessarily lead to an increase in healthy tissue toxicities [33]. This may perhaps be because the decay did not occur within cells or close to radiosensitive subcellular structures.

In this study, gel electrophoresis of plasmid pBR322 was used to systematically relate the DNA damage caused by different radionuclides. This included both co-incubation with the DNA or use as an external source of radiation separate to and relatively distant from the DNA. DNA damage was compared to that caused by EBRT to better understand the RBE of Auger electron-emitters and other radionuclides in a simplified context without the complicating features of living cells and tissues.

## 2 Materials and methods

### 2.1 Radionuclide supply and conversion

[^67^Ga]Ga-citrate (411-430 MBq; 78 h half-life) was obtained in 5.5 mL citrate solution (Mallinckrodt, Netherlands) and converted to [^67^Ga]GaCl_3_ as described previously [34]. In short, [^67^Ga]Ga-citrate was converted with a SEP-PAK® Silica Plus Light cartridge (120 mg sorbent 55-105 μm particle size, Waters). After pre-conditioning the cartridge with 10 mL milliQ water at 1 mL/min, [^67^Ga]Ga-citrate was passed through the cartridge up to three times at 0.5-1 mL/min to maximise retention on the cartridge, which was then washed three times with 5 mL milliQ water. Finally, [^67^Ga]GaCl_3_ was eluted with 600 μL 0.1 MHCl (Advanced Biochemical Compounds, ABX GmbH, Radeberg, Germany) and collected in 50 μL fractions. Fractions with the highest activity (usually fraction 4 or 5) were used for chelator labelling. [^111^In]InCl_3_ (110-200 MBq; 67 h half-life) was supplied in 0.5 mL 0.05 M hydrochloric acid (Mallinckrodt, Netherlands). Radionuclide radiation spectra have been previously published [35].

### 2.2 Plasmid DNA damage

Supercoiled plasmid DNA, pBR322, was obtained from New England Biolabs at 250 μg/mL, in 10 mM Tris-HCl, 1 mM EDTA and pH 8.0. Plasmid DNA, used at 1.25 ng/μL and 5 ng/μL in 20 μL PBS, was irradiated at 0-40 Gy by external beam radiotherapy (EBRT) using a 0.6 MeV gamma-emitting Caesium-137 source (half-life: 30 years) at 5 Gy/min. Separately, plasmid DNA (1.25 ng/μL and 5 ng/μL, 20 μL final volume) was incubated with 0.5-1 MBq [^67^Ga]GaCl_3_ or [^111^In]InCl_3_ for up to 25 h to determine the effect of plasmid concentration, activity and radionuclide on induction of DNA damage. For up to 73 h, plasmid DNA was also incubated with gallium citrate or chelated [^67^Ga]GaCl_3_, i.e. [^67^Ga]Ga-DOTA or [^67^Ga]Ga-THP, to ascertain any protective effects against DNA damage from the chelation of [^67^Ga]GaCl_3_ (0.4-0.5 MBq). [^67^Ga]GaCl_3_ was also used as an external source of radiation by placing a 0.5 mL Eppendorf tube containing plasmid inside a 15 mL centrifuge tube containing 0.5 MBq [^67^Ga]GaCl_3_. Exposure times were chosen to enable us to kinetically model the DNA damage caused by radionuclides. During incubation, samples were kept in the fridge. In the final reaction, the pH was neutral for all conditions.

**Fig 1:**
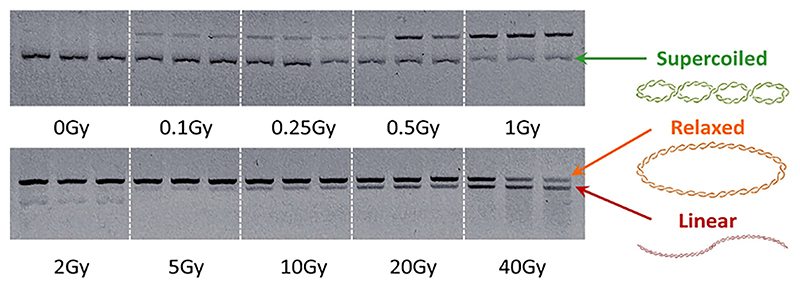
Representative image of triplicate samples of intact, supercoiled plasmid pBR322 irradiated with gamma rays up to a dose of 40 Gy and run on an agarose gel showing the induction of DNA damage as relaxed DNA (i.e. single strand breaks) and linear DNA (i.e. double strand breaks). White lines were superimposed; samples were run on the same gel. Samples at0 Gy show a strong supercoiled band and at 2 Gy a strong relaxed band with faint linear and supercoiled bands.

Plasmid samples (20 μL) were mixed with 4 μL 6× loading dye (ThermoFischer Scientific) and 12 μL of this mixture, i.e. 12.5 or 50 ng plasmid DNA, was run on a 0.8% agarose gel (Fisher Bioreagents) containing GelRed (1:10,000; Sigma Aldrich) at 100 V for 35 min. Agarose gels were imaged after electrophoresis using a GelDoc-ItTS2 310 Imager system, equipped with Benchtop UV transilluminator (UVP) and GelCam 310. Analysis of gel images was performed using ImageJ [36], measuring supercoiled (intact DNA), relaxed circular (single strand breaks) and linear band (double strand breaks) percentages within each lane (Fig. 1). In brief, a rectangle was drawn to define a lane and, the lane was then plotted (with bumps where the bands appear). Using the “Straight” tool, lines were then drawn to define each band and finally, the percentage distribution for each band was calculated.

### 2.3 ^67^Ga chelator radiolabelling

[^67^Ga]GaCl_3_ was chelated with either S-2-(4-isothiocyanatobenzyl)-1,4,7,10-tetraazacyclododecane tetraacetic acid (p-SCN-Bn-DOTA, here referred to as DOTA; Macrocyclics) or (tris)hydroxypyridinone (H3CP256 synthesised by Ma et al. [37], now commercially available as THP from Chematech). DOTA (2.5-5 μg; 35 μL) in 0.25 M ammonium acetate was mixed with [^67^Ga]GaCl_3_ (6-25 MBq; 50 μL; pH 3.5) and heated to 95 °C for 15min. Separately, THP (7 μg; 50 μL) in 0.5M ammonium acetate was mixed with [^67^Ga]GaCl_3_ (26 MBq; 50 μL; pH 3.5) at room temperature for 10 min. In the final reaction, the pH was neutral for all conditions. Radiochemical purity was assessed by instant thin layer chromatography utilising iTLC-SG (glass microfiber chromatography paper impregnated with silica gel; Agilent Technologies, Folsom, USA) giving good separation between [^67^Ga]GaCl_3_ (Rf = 0) and [^67^Ga] Ga-DOTA or [^67^Ga]Ga-THP (Rf = 1) with a mobile phase of 1 M ammonium acetate in water/methanol (1:1; Fig. S1). Strips were analysed with Storage Phosphor films and Cyclone® Plus Storage Phosphor Imager (Perkin-Elmer).

### 2.4 Data analysis and model fitting

Topological conversion rates for supercoiled-to-relaxed, $\left(k_{sr}^{x}\right)$ and relaxed-to-linear, $\left(k_{rl}^{\chi }\right)$, DNA were obtained by fitting a kinetic model to the EBRT, [^67^Ga]GaCl_3_ and [^111^In]InCl_3_ data. The kinetic scheme was assumed to consist of two consecutive irreversible steps

$$S\overset{k_{sr}^{x}}{\to }R\overset{k_{rl}^{x}}{\to }L,$$

where S, R, and *L* are the proportions of supercoiled, relaxed, and linear DNA respectively. Rates are shown as Gy^−1^ (EBRT) or h^−1^ (radionuclides) ± standard error. There is a possibility of an additional transition from S directly to L due to the creation of double strand breaks (DSBs), however, this was deemed unlikely, and we opted for the simplest model that could explain the observations.

Solving the master equations describing this scheme, the proportions of the respective species, as a function of either time or dose, are:

(1) $$S=S_{0}e^{-k_{sr}^{x}x},$$

(2) $$R=S_{0}\frac{k_{sr}^{x}}{k_{rl}^{X}-k_{sr}^{X}}(e^{-k_{sr}^{x}x}-e^{-k_{rl}^{x}x}),$$

(3) $$\text{and}\;L=S_{0}\left(1+\frac{k_{sr}^{x}e^{-k_{rl}^{x}x}-k_{rl}^{X}e^{-k_{sr}^{x}x}}{k_{rl}^{x}-k_{sr}^{\chi }}\right),$$

where *x* is either a given dose, D (Gy), or time, t (h). The data for all three species proportions were fit globally with the above equations using ‘nlinfit’ in custom MATLAB code (Mathworks, 2018b) weighted by the standard deviation. This code is publicly available at github.com/danielburnham/DNA-Damage-Kinetics. With the successful quantitative model in place, it is possible to form the basis of a quantitative comparison between DNA damage created by EBRT and MRT. Briefly, employing the proportion of a specific DNA state as a proxy for DNA damage, we can equate EBRT and MRT-produced damage. Taking the proportion of supercoiled DNA remaining (Eq. (1)) after EBRT and MRT, S_EBRT_(D) and S_MRT_(t) respectively, and equating them we can determine the equivalent dose from EBRT, D, required to achieve the same damage as *t* hours of MRT to be

(4) $$D=\frac{k_{Sr}^{t}}{k_{sr}^{D}}t.$$

Equivalently, the incubation time, *t*, required for a radionuclide at a given activity to achieve the same damage as an EBRT dose, D, was

(5) $$t=\frac{k_{sr}^{D}}{k_{sr}^{t}}D.$$

The same principle was applied to both relaxed and linear states as given in the Supplementary Methods.

### 2.5 PET isotopes

Studies with PET isotopes largely followed the same methods as described above for [^67^Ga]GaCl_3_ and [^111^In]InCl_3_. Here, slight alterations to the method are described in the Supplementary Methods.

### 2.6 Statistical analysis

Plasmid electrophoresis results are shown as mean percentage of total DNA, i.e. supercoiled + relaxed + linear topologies, ±standard deviation. Two-way ANOVA statistical analyses were carried out using Tukey’s multiple comparisons test in GraphPad Prism 7.0c. P < 0.05 was deemed significant. Conversion rates and rate ratios are shown as value ± standard error in the mean.

## 3 Results

### 3.1 Plasmid concentration effect on DNA damage

**Fig 2:**
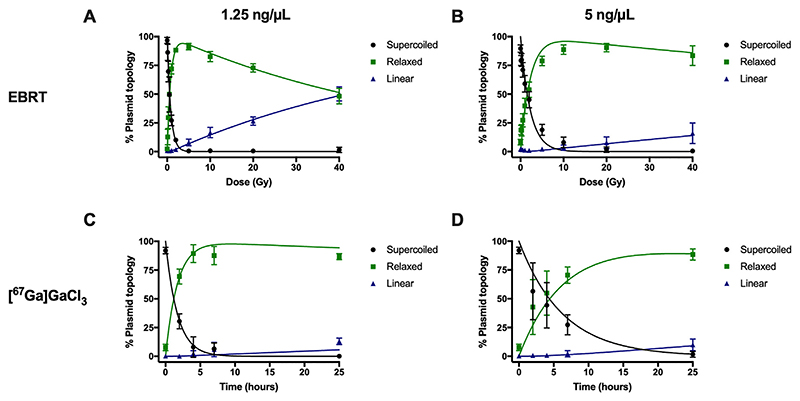
Analysis of 1.25 ng/μL (A, C) and 5 ng/μL (B, D) supercoiled plasmid pBR322 run on agarose gels by electrophoresis. Samples were irradiated by an external beam source (up to 40 Gy; A, B) or incubated with 0.4-0.5 MBq [^67^Ga]GaCl_3_ for up to 25 h (C, D). DNA damage induction was measured through quantification of relaxed DNA (i.e. single strand breaks) and linear DNA (i.e. double strand breaks) relative to supercoiled plasmid (n = 6-22 gel lanes). Superimposed on the biological data points are the modelled fits.

An increasing dose of external beam radiation (EBRT) decreased the percentage of supercoiled plasmid DNA (1.25 ng/μL) from 97 ± 3% (untreated plasmid) to 50 ± 15% and 1.5 ± 2.3% at 0.5 and 40 Gy, respectively (Figs. 1 and 2A). Simultaneously, relaxed plasmid DNA (single strand breaks) increased from 2 ± 2% to 50 ± 15% and 48 ± 7% at 0.5 and 40 Gy, whilst linear DNA (double strand breaks) increased from 0.9 ± 0.6% to 50 ± 6% at 40 Gy. An increase in plasmid concentration (5 ng/μL) that was subsequently irradiated affected the relative percentage of relaxed and linear DNA in the sample (Fig. 2B). For example, for 5 ng/μL of plasmid, a higher percent of supercoiled DNA was observed at 0.5 Gy (71 ± 6%) than for 1.25 ng/μL plasmid DNA (2-way ANOVA between values at 1.25 and 5 ng/μL; P < 0.0001). This observation correlates to changes in relative percentages of relaxed and linear plasmid DNA, with a slower induction of relaxed DNA (P = 0.0124; comparison between relaxed DNA at 1.25 and 5 ng/μL) and a lower conversion of this relaxed DNA to linear DNA (P < 0.0001; comparison between linear DNA at 1.25 and 5 ng/μL) at the higher plasmid concentration. For example, at 5 ng/μL, relaxed DNAforms comprised 27 ± 6% at 0.5 Gy, whereas only 16 ± 9% was found for linear DNA at 40 Gy.

In MRT, an increase in radiation dose can be delivered by increasing the incubation time of cells with the radionuclide. As such, increasing the incubation time of plasmid DNA with [^67^Ga]GaCl_3_ also decreased the relative amount of supercoiled DNA from 92 ± 3% (0 h incubation) to 31 ± 7% to 0.14 ± 0.05% at 2 and 25 h, respectively (Fig. 2C). The presence of relaxed DNA increased from 8 ± 3% to 70 ± 7% and 87 ± 3% at 2 and 25 h, respectively, whereas linear DNA was quantified at 0.2 ± 0.3%, 0.11 ± 0.05% and 13 ± 3% at 0, 2 and 25 h, respectively.

Similar to EBRT, a lower percentage of overall damage was observed at higher concentrations of the plasmid when incubated with [^67^Ga] GaCl_3_ (Fig. 2D). For example, for 5 ng/μL of plasmid, a higher percentage of supercoiled DNA was observed at 2 and 25 h (57 ± 25% and 2 ± 3%, respectively) than for 1.25 ng/μL plasmid DNA (P = 0.001). This observation was also correlated to a change in the relative amounts of relaxed plasmid DNA, with less DNA relaxation evident after 2 h (43 ± 24%; P < 0.0005; comparison between relaxed DNA at 1.25 and 5 ng/μL). In contrast, no change in the generation of linear DNA was observed upon increasing plasmid concentrations from 1.25 ng/μL to 5 ng/μL (P = 0.1560).

### 3.2 [^67^Ga]GaCl_3_ chelator radiolabelling

The labelling efficiencies for DOTA and THP with [^67^Ga]GaCl_3_ were 91–98% and 88–91%, respectively (Fig. S1). This led to specific activities of 1–10 MBq/μg (0.5–5 MBq/nmol DOTA) and 3-3.5 MBq/μg (3–3.5 MBq/nmol THP) for [^67^Ga]Ga-DOTA and [^67^Ga]Ga-THP, respectively.

### 3.3 DNA damage by chelated [^67^Ga]GaCl_3_

**Fig 3:**
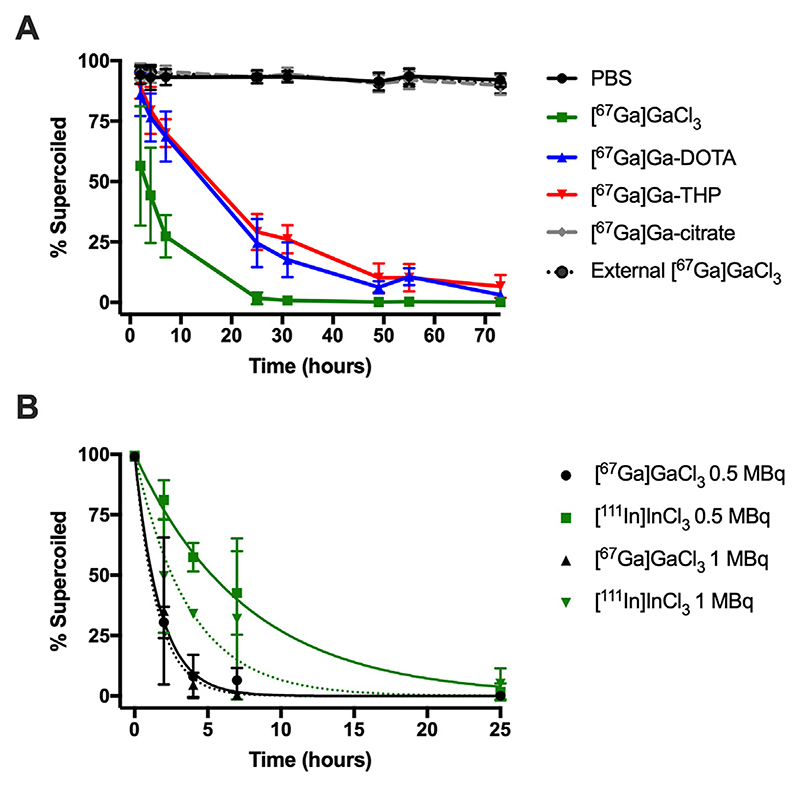
Analysis of percentage of supercoiled plasmid pBR322 DNA run on agarose gels by electrophoresis. (A) Plasmid DNA (5 ng/μL) was incubated with 0.4-0.5 MBq [^67^Ga] GaCl_3_, [^67^Ga]GaCl_3_ chelated with DOTA or THP, [^67^Ga]Ga-citrate or [^67^Ga]GaCl_3_ as an external radiation source for up to 73 h (n = 9-22 gel lanes). Data were not modelled to determine rates of conversion. (B) Plasmid DNA (1.25 ng/μL) was incubated with 0.5 or 1 MBq [^67^Ga]GaCl_3_ or [^111^In]InCl_3_ for up to 25 h (n = 3-9). Superimposed on the biological data points in (B) are the model fits. The standard deviation bar at 4 h for 1 MBq [^111^In]InCl_3_ is too small to be visualised on the graph.

The effect on plasmid DNA damage induction by chelated [^67^Ga] GaCl_3_ was measured by monitoring the decrease in the relative levels of supercoiled DNA (Fig. 3A). Chelation of [^67^Ga]GaCl_3_ by DOTA or THP led to a higher level of intact DNA at 25 ± 10% or 29 ± 8%, respectively, compared to plasmid DNA treated with unchelated [^67^Ga]GaCl_3_ (2 ± 3%, P < 0.0001) at 25 h. There was no significant difference found between values obtained for [^67^Ga]Ga-DOTA or [^67^Ga]Ga-THP (P = 0.09).

Separately, a preventative effect was also observed for [^67^Ga]Ga-cit-rate as well as when [^67^Ga]GaCl_3_ was used as an external source of radiation, i.e. not in the same tube as the plasmid (P < 0.0001).

### 3.4 DNA damage induced by [^67^Ga]GaCl_3_ versus [^111^In]InCl_3_

Both [^67^Ga]GaCl_3_ and [^111^ln]lnCl_3_ Auger electron-emitters reduced the level of supercoiled plasmid DNA over time (Fig. 3B). [^67^Ga]GaCl_3_ proved highly damaging with little difference in damage induced when plasmid DNA was incubated with either 0.5 or 1 MBq (P = 0.8026). On the other hand, increasing the activity from 0.5 to 1 MBq for [^111^In]InCl_3_ did decrease the level of supercoiled DNA from 81 ± 8% to 50 ± 24% at 2 h (P = 0.0299). Furthermore, [^67^Ga]GaCl_3_ proved to be more damaging than [^111^In]InCl_3_ (P < 0.0001 at 0.5 MBq and P = 0.0140 at 1 MBq). For example, supercoiled DNA levels were at 31 ± 7% and 81 ± 8% for [^67^Ga]GaCl_3_ and [^111^In]InCl_3_, respectively at 0.5 MBq at 2 h.

### 3.5 Model fitting

**Fig 4:**
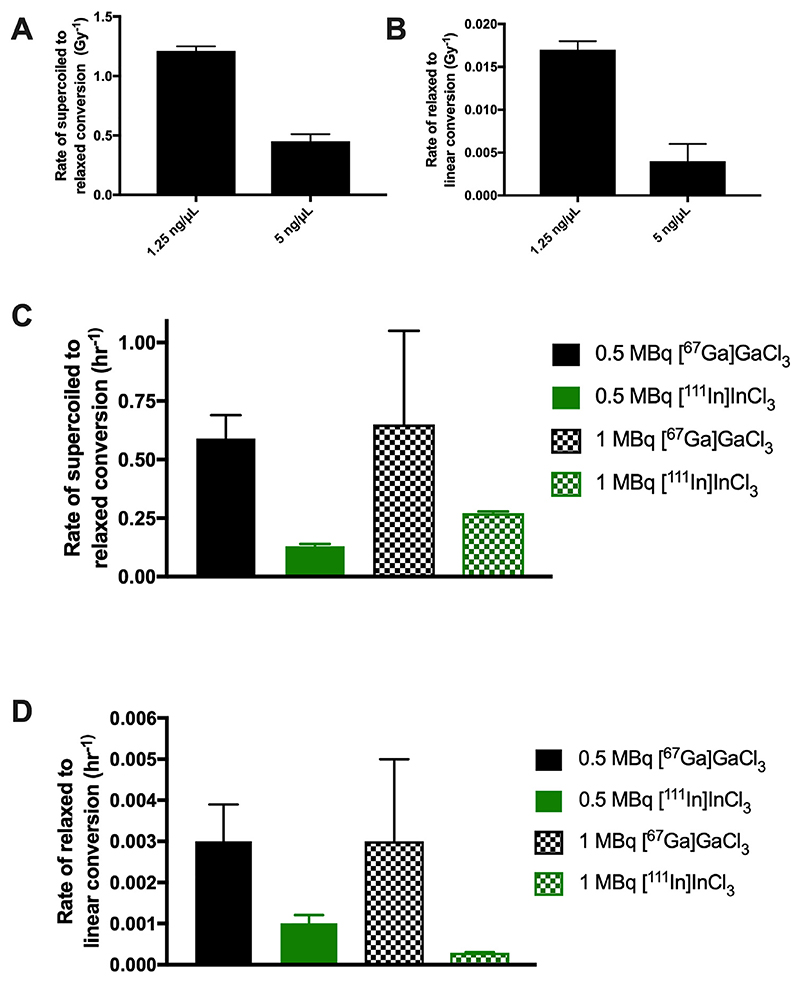
Rates of conversion of 1.25 ng/μL or 5 ng/μL supercoiled pBR322 plasmid irradiated by external beam radiotherapy (A, B) or 1.25 ng/μL plasmid incubated with 0.5 or 1 MBq [^67^Ga] GaCl_3_ or [^111^In]InCl_3_ (C,D). Figs depict values obtained for plasmid DNA conversion from supercoiled DNA to relaxed DNA, i.e. from intact DNA to single strand breaks $\left(k_{sr}^{x}\right)$ or plasmid DNA conversion from relaxed DNA to linear DNA, i.e. from single to double strand breaks $(k_{rl}^{\chi }).$

Fitting a kinetic model to the biologically obtained plasmid data enabled the calculation of topological conversion rates from supercoiled to relaxed DNA $\left(k_{sr}^{x}\right)$ and relaxed to linear DNA $\left(k_{rl}^{\chi }\right)$ (Fig. 4). Topological conversion rates were faster for plasmid DNA irradiated by EBRT at concentrations of 1.25 ng/μL($k_{sr}^{D}$ 1.21 ±0.04 Gy^−1^; $k_{rl}^{D}$0.017 ±0.001 Gy^−1^) compared to 5 ng/μL ($k_{sr}^{D}$ 0.45 ± 0.06 Gy^−1^; $k_{rl}^{D}$ 0.004 ± 0.002 Gy^−1^) (Fig. 4A–B, Table S1). Similarly, topological conversion rates from supercoiled to relaxed were faster for plasmid DNA incubated with [^67^Ga]GaCl_3_ at a concentration of 1.25 ng/μL ($k_{sr}^{t}$0.590 ± 0.100 h^−1^) compared to 5 ng/μL ($k_{sr}^{t}$ 0.159 ± 0.009 h^−1^) (Tables S2–3). Through developing a kinetic understanding of the mechanism and data, we can determine a difference between 1.25 and 5 ng/μl, increasing our understanding, that would otherwise have remained hidden. In all cases $k_{sr}^{x}$ > $k_{rl}^{\chi }$ thus the second kinetic step, is rate-limiting, with the implication that DSBs are formed by the appearance of closely neighbouring single strand breaks.

The rate of conversion of supercoiled to relaxed DNA was higher for [^67^Ga]GaCl_3_ (0.59 ± 0.10 h^−1^ at 0.5 MBq) than for [^111^In]InCl_3_ (0.13 ± 0.01 h^−1^ at 0.5 MBq). This was also the case for the rate of conversion of relaxed to linear DNA with values of 0.003 ± 0.001 h^−1^ for [^67^Ga] Gacl_3_ and 0.0010 ± 0.0002 h^−1^ for [^111^In]InCl_3_ at 1.25 ng/μL (Fig. 4C–D). In all studies, the negative control consisting of untreated plasmid DNA did not show evidence of damage over the corresponding timeframe within the errors associated with the measurement (Fig. S5).

All calculated rates and respective uncertainties are given in Supplementary Tables 1–3.

### 3.6 Comparing DNA damage from EBRT and [^67^Ga]GaCl_3_

Having measured the damage caused by EBRT and MRT and calculated the rates of transition between different DNA damaged states using Eqs. (2)–(3), we can now use Eq. (4) to calculate the equivalent EBRT dose for a given time of radionuclide incubation, producing the same DNA damage. Equivalently, we can calculate the time needed to incubate with a given radionuclide to obtain the same damage caused by a certain EBRT dose (Eq. (5)).

**Fig 5:**
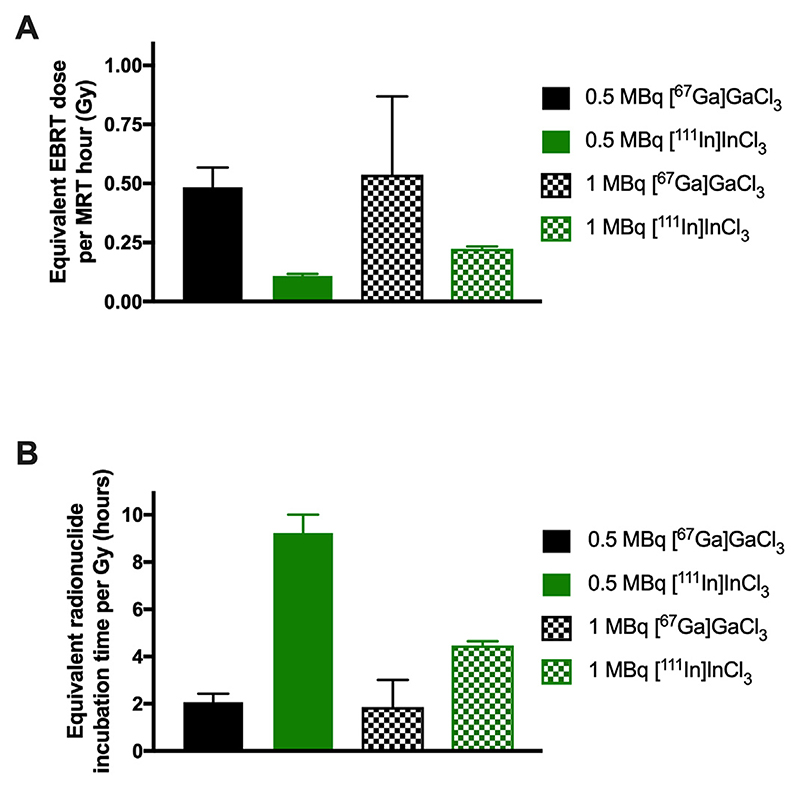
(A) Calculated EBRT dose (Gy) that induces the same amount of DNA damage (from supercoiled to relaxed DNA) as after 1 h incubation of the plasmid with 0.5 or 1 MBq of [^67^Ga]GaCl_3_ or [^111^In]InCl_3_ (ratio from Eq. (4)). (B) Calculated incubation time for 0.5 or 1 MBq [^67^Ga]GaCl_3_ or [^111^In]InCl_3_ that induces the same amount of DNA damage (from supercoiled to relaxed DNA) as 1 Gy EBRT (ratio from Eq. (5)). Values in both graphs have been calculated for 1.25 ng/μL plasmid DNA.

To elucidate the principle, we now consider some examples using Eq. (4) and the rates from Tables S1–3 Fig. 5). When 1.25 ng/μL plasmid DNA was incubated with 0.5 MBq [^67^Ga]GaCl_3_ for 1 h, the equivalent dose is found to be D_Ga_ = (0.585 ± 0.1 h^−1^ / 1.21 ± 0.04 Gy^−1^) × 1 h = 0.48 Gy ± 0.08 Gy, and for [^111^In]InCl_3_ under the same conditions D_In_ = (0.131 ± 0.01 h^−1^ / 1.21 ± 0.04 Gy^−1^) × 1 h = 0.11 Gy ± 0.01 Gy. Similarly, incubating plasmid DNA with 1 MBq [^67^Ga]GaCl_3_ or [^111^In]InCl_3_ for 1 h induces that same amount of DNA damage as 0.5 ± 0.3 Gy and 0.224 ± 0.009 Gy of EBRT, respectively.

Alternatively, using Eq. (5) and the rates from Tables 1–3, for 1.25 ng/μL plasmid DNA irradiated at 1 Gy, the equivalent incubation time with 0.5 MBq [^67^Ga]GaCl_3_ is found to be, t_Ga_ = (1.21 ± 0.04 Gy^−1^ / 0.585 ± 0.1 h^−1^) × 1 Gy = 2.05 ± 0.36 h and for [^111^In]InCl_3_ under the same conditions t_In_ = (1.21 ± 0.04 Gy^−1^ / 0.131 ± 0.01 h^−1^) × 1 Gy = 9.3 ± 0.77 h (Fig. 5, Tables S1–3).

### 3.7 PET isotopes

Incubation of plasmid DNA with PET isotopes 0.5 MBq [^52^Mn]MnCl_2_, 0.5 MBq [^89^Zr]Zr-oxine, and 1-5 MBq [^68^Ga]GaCl_3_ also caused relaxation of the DNA (Figs. S2–4). Although no double strand breaks were formed after 12 days, single stranded breaks were induced at 6 days when [^52^Mn]MnCl_2_ was used as an external source (Fig. S2). Similarly, [^89^Zr] Zr-oxalate, when used as an external source of radiation, induced single and double strand breaks at day 3 of incubation, whereas the [^89^Zr]Zr-oxine co-incubated with the plasmid DNA or control groups did not cause damage (Fig. S3A). The amount of single and double strand DNA breaks increased between day 3 and 7 for external [^89^Zr]Zr-oxalate (Fig. S3B).

## 4 Discussion

Here, studies were carried out that systematically compared the effects of radiation from radionuclides with radiation from EBRT in a cell-free environment with plasmid DNA as a probe. This follows on from previous plasmid-based work that determined the ability of Auger electron-emitting radionuclides to induce SSBs and DSBs and their dependence on the DNA’s topology or proximity [13,14,19,20,22,38–41]. Previous studies were mainly focussed on the Auger electron-emitting radionuclides iodine-123 and iodine-125. In silico simulation studies have also been reported, demonstrating that the number of DSBs induced by Auger electron-emitters and other MRT radionuclides depends on their proximity to the DNA as well as the DNA form, e.g. B-form [42,43].

Only few prior reports employing plasmids as DNA probes directly compared radionuclides with EBRT. For example, the mean lethal dose of radiation (D_0_; dose that decreases intact plasmid DNA by 67%), was calculated to be 3.1 ± 0.1 Gy and 2.8 ± 0.1 Gy for an external ^137^Cs source (+DTPA) and DTPA-chelated [^111^In]InCl_3_, respectively [40]. [^111^In]InCl_3_ was most effective in the absence of DTPA (i.e. when up to 15% was bound to DNA) with a D_0_ value of 15.3 + 0.7 × 10^10^disintegrations/cm^3^ [40]. Similarly, gamma irradiation from Caesium-137 led to an exponential decrease in the presence of supercoiled plasmid DNA with a D_0_ value of 10.8 ± 0.3 Gy. Under identical conditions, the D_0_ values for an iodine-125 labelled radiopharmaceutical that intercalated the DNA, 2-[^125^I]iodoacridine, and a non-intercalating equivalent, 4-[^125^I]iodoacridine, were 22.4 ± 0.6 × 10^11^ disintegrations and 4.7 ± 0.4 × 10^11^ disintegrations, respectively [22].

The work presented here demonstrates how the cell-free plasmid pBR322 study can be used to not only compare the biological effects of various radionuclides with one another and with the effects from EBRT, but to also provide an easy, quantitative comparison between radiation absorbed dose from EBRT (Gy) and time of radionuclide incubation at different activities (MBq). For example, the rate of damage was greater for 0.5 and 1 MBq [^67^Ga]GaCl_3_ than [^111^In]InCl_3_. This differs from in silico simulations that predicted that [^111^In]InCl_3_ created more SSBs and DSBs than [^67^Ga]GaCl_3_ [43] and demonstrates that understanding the mechanism and making fundamental measurements of DNA damage kinetics, as well as DNA binding capacity of radionuclides, will help to refine future in silico models and simulations.

Here, we also show the dependence of the DNA damaging effects from radionuclides and EBRT on plasmid concentration used. For example, both rates of conversion from supercoiled to relaxed DNA (single strand breaks; $\left(k_{sr}^{x}\right)$ and from relaxed DNA to linear DNA $\left(k_{rl}^{\chi }\right)$ decreased when a higher concentration of plasmid (5 ng/μL, 100 ng) was present during irradiation by EBRT. Also, $k_{rl}^{\chi }$ was slower than $k_{sr}^{x}$, probably due to an increased likelihood in creating single strand breaks than double strand breaks per decay. DSBs are more likely to be formed from two single strand nicks within a few bases of each other. Similarly, the rate of conversion from supercoiled DNA to relaxed DNA also decreased when a higher concentration of plasmid was incubated with 0.5 MBq [^67^Ga]GaCl_3_. These data thus show that the plasmid assay could provide useful mechanistic insights into DNA damage caused by radionuclides. Given the similarities between the effects of increasing plasmid concentration for both EBRT and MRT, we propose that there is a common mechanism at work. Also, it highlights the need for a systematic approach to maximise output and validity of results as values such as D_0_ [22,40] and RBE [44] obtained in studies in which different plasmid concentrations were used cannot be compared.

Also, as shown for other Auger electron-emitters, the amount of DNA damage induced by [^67^Ga]GaCl_3_ was dependent on its proximity to the DNA, since the chelation of [^67^Ga]GaCl_3_ by DOTA and THP, resulting in the formation of negatively and uncharged complexes, respectively, decreased the amount of DNA damage as compared to the ionic gallium salt, [^67^Ga]GaCl_3_. Not only does chelation result in steric hindrance, preventing the positively charged radioisotope from directly interacting with DNA, but it also affects the net molecular charge of the radioactive species, from +3 [^67^Ga]Ga to –1 for [^67^Ga]Ga-DOTA and neutral (0) for [^67^Ga]Ga-THP. The change in charge likely results in reduced, or even repulsive (for ([^67^Ga]Ga-DOTA)^1−^) forces between the radioactive species and the negatively charged DNA backbone. The use of [^67^Ga]Ga-citrate, which has a charge of−5, or [^67^Ga]GaCl_3_ as an external source of radiation proved to completely protect the DNA against damage from [^67^Ga] Ga^3+^, as seen previously [19]. Unfortunately, it is unclear in these studies how much of the radionuclides bound to the plasmid DNA.

In these studies, as described elsewhere [45], it was deemed important to investigate the damage caused by radionuclides when used as an external source (i.e. not sharing the same solution or microscopic space), to look at damage caused not by low energy electron (which are absorbed by the tube material), but instead longer ranging gamma rays, or in the case of PET radionuclides, positrons (which have some penetration through the tube material). Previously, it has been shown that PET isotopes can result in phosphorylation of histone H2AX, a proxy for double strand break induction [46,47]. As such, we also carried out pBR322 DNA damage studies with PET imaging isotopes, which showed [^52^Mn]MnCl_2_, [^89^Zr]Zr-oxalate, and [^68^Ga]GaCl_3_ could induce damage (Figs. S2–4). Whether the latter is due to the AEs emitted from [^68^Ga]GaCl_3_ [48] is currently unexplored. This demonstrated the translatability of this assay to assess the DNA damaging potential of radionuclides used in PET imaging. Equally, the work here further highlights the differences between EBRT and radionuclides in terms of their ability to damage DNA through indirect mechanisms, i.e. water radiolysis, through which the majority of DNA damage is caused during EBRT. We previously showed that DNA damage was reduced for both gallium-67 and indium-111 when plasmid DNA was co-incubated with reactive oxygen scavenger DMSO, although the majority of the damage was caused through direct mechanisms [19].

From Fig. 3B, it can be noted that the minimum and maximum sensitivity of the pBR322 assay does come into play even for radionuclides, as the damage caused by 0.5 MBq [^67^Ga]GaCl_3_ is not different from that caused by 1 MBq; indeed the rates are equal within the associated uncertainty. Either the sensitivity of the assay is insufficient to discriminate the damage by 0.5 and 1 MBq or some type of threshold damage level has been reached; the former of which seems more likely.

Fitting a kinetic model has not previously been presented alongside biological pBR322 study data and yet it is a potentially powerful tool. Here, we assume an irreversible, three state model to explain the observations, however, it is likely an additional irreversible step exists from supercoiled to linear DNA due to single strand breaks occurring sufficiently close to linearise the DNA. This additional kinetic rate, thus model free parameter, does not change the general solution to the master equations of the kinetic scheme, rather only including the addition of a constant to k_sr_ (becoming k_sr_ + k_sl_). Given the observables of our experiments, we are unable to distinguish between these two models and a resolution is for future studies. Also, as seen in Fig. 1, at high levels of DNA damage, e.g. 40 Gy, the measurement of the amount of linear DNA present becomes unreliable, due to the creation of different sized linear fragments, visible both as a distinct band and a smear on the gel.

In this study, kinetic modelling has enabled us to determine the duration of time required to incubate with [^67^Ga]GaCl_3_ or [^111^In]InCl_3_ to induce the same amount of plasmid DNA damage as a specific amount of absorbed radiation dose by EBRT (and vice versa). Traditionally, in vitro or preclinical RBE values are based on clonogenic survival data or studies looking at spermatogenesis in mouse testes [49–51]. These values are then used to inform radiation weighting doses, which in turn are applied to calculate the radiation equivalent dose and finally the effective dose. Yet, it is well known that the RBE values are not accurate for Auger electron-emitters due to uncertainties about subcellular localisation of the radionuclides and unhelpful assumptions about homogeneous distribution of a radionuclide within individual cells and amongst multiple cells [49]. This reliance of biological effect from Auger electron-emitters on their close localisation to the DNA has led to a lack of uptake of relevant radiation weighting values, which have been proposed to be as high as 20 for stochastic effects, by the International Commission on Radiological Protection (ICRP) [52,53]. The observation that chelation can have a marked effect on DNA damage even with intimate mixing of plasmids and radionuclides suggests that weighting factors could be grossly misleading in the case of AE MRT.

As such, it remains to be seen whether this cell-free pBR322 assay will inform RBE and, in turn, affect radiation weighting doses in the clinic. However, this straightforward, cell-free assay with which we can determine cell-free RBE does hold promise and merit in preclinical nuclear imaging/MRT-focussed research groups, in order to better inform choices of ‘safe’ imaging radionuclides and effective therapeutic radionuclides. These data also highlight the gross gaps in understanding that is attainable by conventional dosimetry and weighting factors and how this assay could provide a way to improve that understanding.

## 5 Conclusion

This work demonstrated the utility of the plasmid DNA assay for rapid assessment of the relative effects of medical radionuclides on DNA integrity. This is important for the evaluation of the safety and therapeutic efficacy of radioisotopes used in radionuclide imaging and/or MRT research and to compare them relative to one another and in relation to EBRT. This assay is of particular importance for assessing Auger electron-emitting radioisotopes and to further inform radiopharmaceutical design and in vitro and in vivo therapeutic studies.

## Supplementary Material

**Appendix A. Supplementary data**

Supplementary data to this article can be found online at https://doi.org/10.1016/j.nucmedbio.2021.06.004.

Supplementary data
