# Supplementary material for: Validation of the plasmid study to relate DNA damaging effects of radionuclides to those from external beam radiotherapy
Source: Nucl Med Biol. Author manuscript; Available in PMC 2021 Sep 19. (PMC7611685; doi:10.1016/j.nucmedbio.2021.06.004)
Supplement: Supplementary data [file EMS134946-supplement-Supplementary_data.docx]

**SUPPLEMENTARY FILES**

**Dose-time and time-dose conversion rates for relaxed and linear DNA proportions:**

$\boldsymbol{D= -}\frac{\boldsymbol{1}}{\boldsymbol{k}_{\boldsymbol{rl}}^{\boldsymbol{D}}}\text{ln}\boldsymbol{[}\boldsymbol{e}^{\boldsymbol{-}\boldsymbol{k}_{\boldsymbol{rl}}^{\boldsymbol{t}}\boldsymbol{t}}\boldsymbol{+R}\left( \frac{\boldsymbol{k}_{\boldsymbol{rl}}^{\boldsymbol{t}}}{\boldsymbol{k}_{\boldsymbol{sr}}^{\boldsymbol{t}}}\boldsymbol{-}\frac{\boldsymbol{k}_{\boldsymbol{rl}}^{\boldsymbol{D}}}{\boldsymbol{k}_{\boldsymbol{sr}}^{\boldsymbol{D}}} \right)\boldsymbol{]}$

**t =** $\boldsymbol{-}\frac{\boldsymbol{1}}{\boldsymbol{k}_{\boldsymbol{rl}}^{\boldsymbol{t}}}\mathbf{ln}\boldsymbol{[}\boldsymbol{e}^{\boldsymbol{-}\boldsymbol{k}_{\boldsymbol{rl}}^{\boldsymbol{D}}\boldsymbol{D}}\boldsymbol{- R}\left( \frac{\boldsymbol{k}_{\boldsymbol{rl}}^{\boldsymbol{t}}}{\boldsymbol{k}_{\boldsymbol{sr}}^{\boldsymbol{t}}}\boldsymbol{-}\frac{\boldsymbol{k}_{\boldsymbol{rl}}^{\boldsymbol{D}}}{\boldsymbol{k}_{\boldsymbol{sr}}^{\boldsymbol{D}}} \right)\boldsymbol{]}$

$$\boldsymbol{D= -}\frac{\boldsymbol{1}}{\boldsymbol{k}_{\boldsymbol{rl}}^{\boldsymbol{D}}}\mathbf{ln}\left[ \left( \boldsymbol{1-}\frac{\boldsymbol{k}_{\boldsymbol{sr}}^{\boldsymbol{t}}}{\boldsymbol{k}_{\boldsymbol{rl}}^{\boldsymbol{t}}} \frac{\boldsymbol{k}_{\boldsymbol{rl}}^{\boldsymbol{D}}}{\boldsymbol{k}_{\boldsymbol{sr}}^{\boldsymbol{D}}} \right)\left( \boldsymbol{1-L} \right)\boldsymbol{+}\frac{\boldsymbol{k}_{\boldsymbol{sr}}^{\boldsymbol{t}}}{\boldsymbol{k}_{\boldsymbol{rl}}^{\boldsymbol{t}}} \frac{\boldsymbol{k}_{\boldsymbol{rl}}^{\boldsymbol{D}}}{\boldsymbol{k}_{\boldsymbol{sr}}^{\boldsymbol{D}}} \boldsymbol{e}^{\boldsymbol{-}\boldsymbol{k}_{\boldsymbol{rl}}^{\boldsymbol{t}}\boldsymbol{t}} \right]$$

$$\boldsymbol{t = -}\frac{\boldsymbol{1}}{\boldsymbol{k}_{\boldsymbol{rl}}^{\boldsymbol{t}}}\mathbf{ln}\left[ \left( \boldsymbol{1-}\frac{\boldsymbol{k}_{\boldsymbol{sr}}^{\boldsymbol{D}}}{\boldsymbol{k}_{\boldsymbol{rl}}^{\boldsymbol{D}}} \frac{\boldsymbol{k}_{\boldsymbol{rl}}^{\boldsymbol{t}}}{\boldsymbol{k}_{\boldsymbol{sr}}^{\boldsymbol{t}}} \right)\left( \boldsymbol{1-L} \right)\boldsymbol{+}\frac{\boldsymbol{k}_{\boldsymbol{sr}}^{\boldsymbol{D}}}{\boldsymbol{k}_{\boldsymbol{rl}}^{\boldsymbol{D}}} \frac{\boldsymbol{k}_{\boldsymbol{rl}}^{\boldsymbol{t}}}{\boldsymbol{k}_{\boldsymbol{sr}}^{\boldsymbol{t}}} \boldsymbol{e}^{\boldsymbol{-}\boldsymbol{k}_{\boldsymbol{rl}}^{\boldsymbol{D}}\boldsymbol{D}} \right]$$

**PET isotopes methodology**

Plasmid DNA damage studies with PET isotopes largely followed the same method as described in the main text for [^67^Ga]GaCl_3_ and [^111^In]InCl_3._ Here, slight alterations to the method are described for:

(i) Manganese-52: 100 ng of plasmid pBR322 (0.1 μg/μL) was placed in a 0.2 mL tubes designed for polymerase chain reactions (PCRs). One tube was made up to 30 μL by the addition of sterile phosphate-buffered saline (PBS; pH 7.4) to form the negative control. Another tube was made up to 30 μL in PBS, and placed in a larger tube containing 0.5 MBq [^52^Mn]MnCl_2_ (40 mL, enough to cover the bottom 30 μL of the PCR tube). To the final tube was added neutralised 0.5 MBq [^52^Mn]MnCl_2_ (adjusted to pH 7). The final tube was also made up to 30 μL by the addition of PBS (pH 7.4). At the relevant timepoint (6 and 13 days; approximately equivalent to one and two half-lives of manganese-52, respectively), 15 μL of the plasmid-containing solution was used for gel electrophoresis. This experiment was performed in duplicate.

(ii) Zirconium:-89: ^89^Zr was acquired as an oxalate salt solution from Perkin-Elmer (Netherlands) typically at a radioactive concentration of 1 MBq/μl. The solution was neutralised to pH 7.5-8 using 1 M Na_2_CO_3_ and the volume increased to 10-fold using dH_2_O. For chelation to 8-hydroxyquinoline (oxine), oxine was dissolved in chloroform to a 10 mg/ml concentration and added to the neutralised ^89^Zr-oxalate solution and shaken for 5 minutes for solvent extraction. The level of chloroform was then increased to 500 μl and the mixture shaken for a further 5 minutes. After shaking, the lower layer (chloroform layer) was removed to a glass Wheaton vial and the amount of radioactivity extracted from the aqueous layer recorded to determine the yield of the reaction. The chloroform was evaporated at 65°C under nitrogen flow and re-dissolved in 30 μl ethanol and 20 μl of PBS by slowly adding each liquid to avoid precipitation. Radio-iTLC (instant thin layer chromatography) was carried out using silica gel impregnated iTLC strips as the solid phase and ethyl acetate as the mobile phase to ensure >95% labelling of oxine with zirconium-89. The same procedure was observed without ^89^Zr in order to prepare the 8-hydroxyquinoline control for plasmid studies (50 ng plasmid). 0.5 MBq of ^89^Zr-oxine was added to the plasmid for 3 or 7 days. Other control samples included PBS and 1 M oxalate with each sample run in triplicate. Also, externally irradiated plasmid samples were created by placing 100 ng of plasmid (10 μl plasmid solution) in a PCR tube and placing this (in triplicate) in a 50 ml conical tube containing 0.5 MBq of ^89^Zr-oxalate in 50 μl dH_2_O thus creating samples in which the radioactivity is external to the plasmid solution, separated by the PCR tube’s plastic wall. (N=3).

(iii) Gallium-68: pBR322 plasmid DNA was incubated with 1 or 5 MBq [^68^Ga]GaCl_3_ for 0.5, 3 and 6 hours (n=2, triplicate technical repeats). Plasmid-containing solution was used for gel electrophoresis to determine effects of gallium-68 on the integrity of supercoiled DNA.


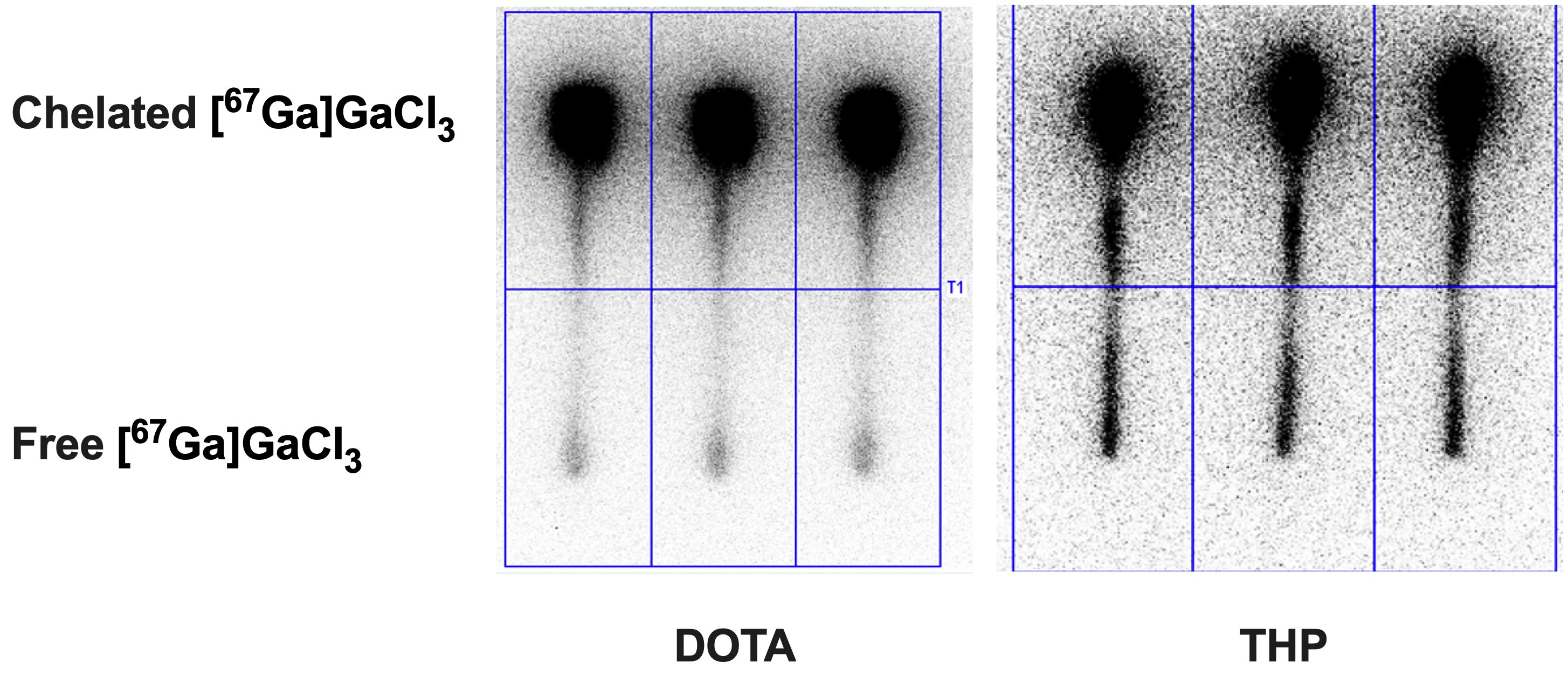


**Figure S1.** ITLC chromatograms of DOTA (left) and THP (right) chelators radiolabelled with [^67^Ga]GaCl_3_ (in triplicate).


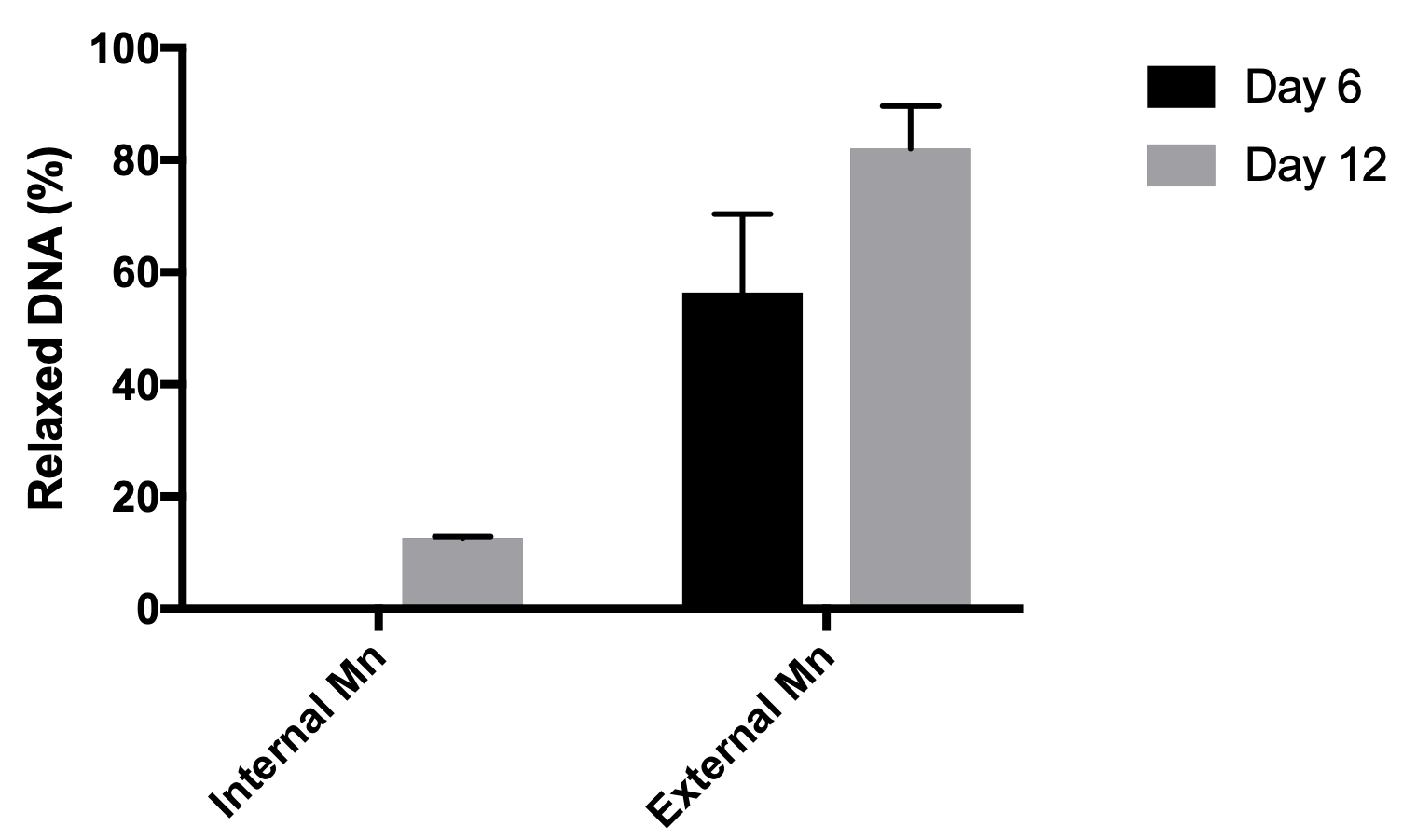


**Figure S2.** Analysis of pBR322 plasmid DNA damaged to the relaxed form of DNA, i.e. single strand breaks, after incubation with 0.5 MBq [^52^Mn]MnCl_2_ for 6 or 12 days. Plasmid was either incubated with [^52^Mn]MnCl_2_ directly (internal) or in a separate tube where [^52^Mn]MnCl_2_ served as an external source of radiation (external) (n=2 because of limited radionuclide availability).


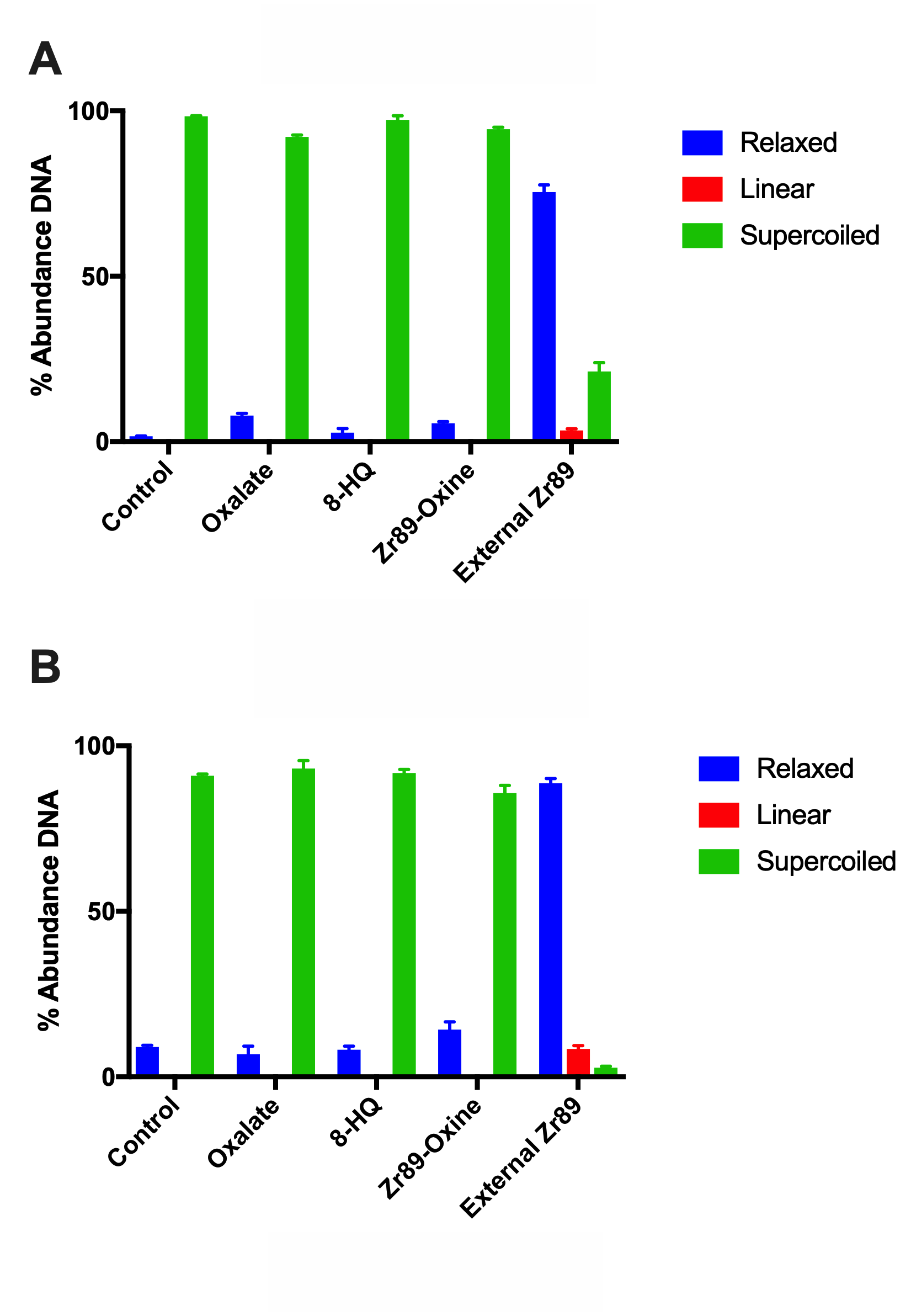


**Figure S3.** Analysis of pBR322 plasmid DNA damaged to the relaxed form of DNA, i.e. single strand breaks, and linear form of DNA, i.e. double strand breaks, after incubation with 0.5 MBq [^89^Zr]Zr-oxine for 3 **(A)** or 7 days **(B)**. Plasmid was either incubated with PBS (control), non-radioactive oxalate (control with oxalic acid in which zirconium-89 arrives), oxine only (8-HQ; 8-Hydroxyquinoline), or [^89^Zr]Zr-oxalate placed in a separate tube to the plasmid where it served as an external source of radiation (external ^89^Zr). N=3 per group.


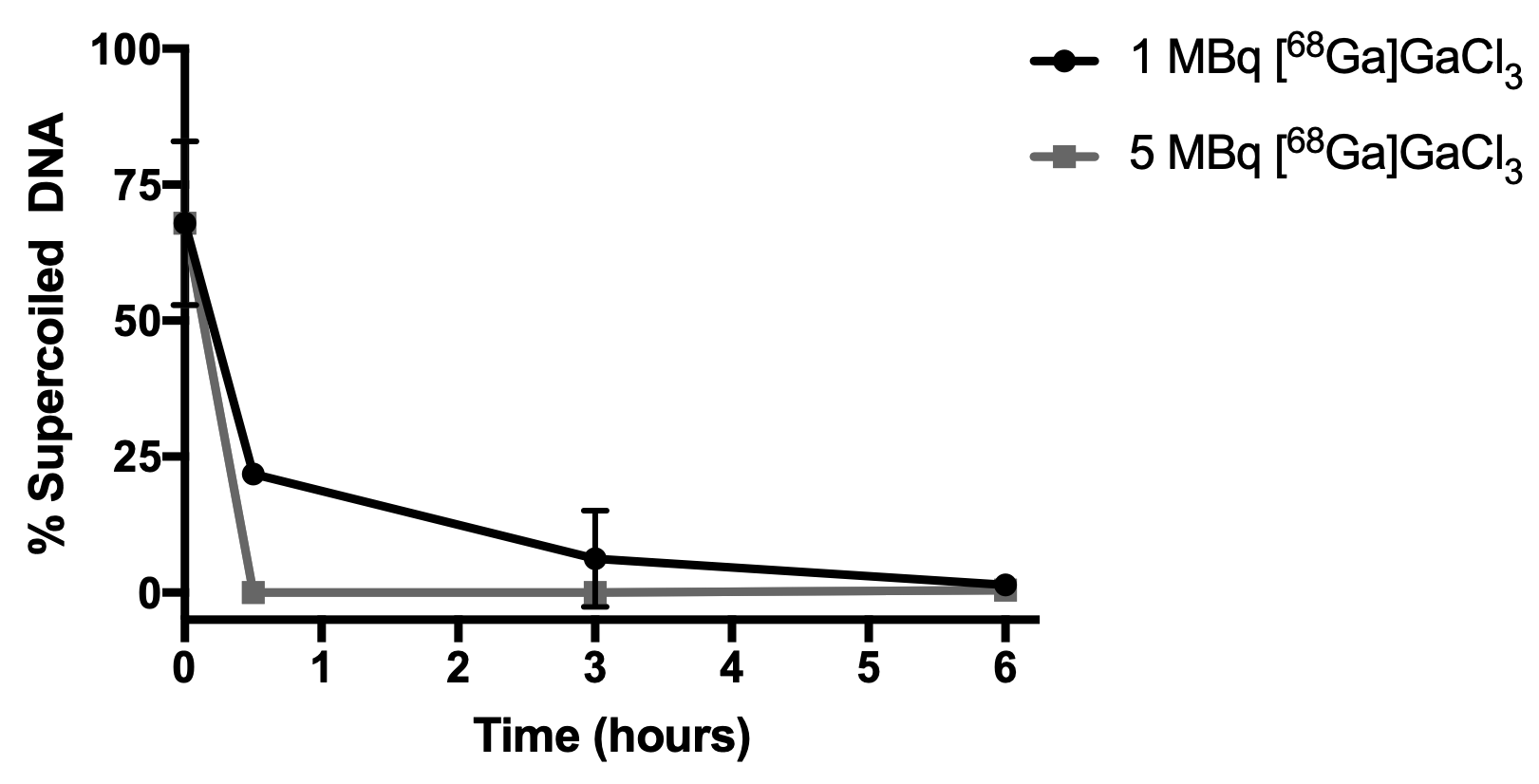


**Figure S4.** Analysis of pBR322 plasmid DNA damaged to the relaxed form of DNA, i.e. single strand breaks, after incubation with 1 or 5 MBq [^68^Ga]GaCl_3_ for 0.5, 3 and 6 hours. (n=2 with 3 technical repeats).


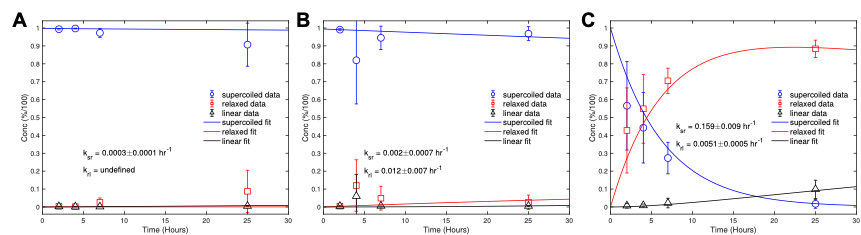


**Figure S5**. Kinetic modelling of damage accumulated after 1.25 ng/µL **(A)** or 5 ng/µL **(B)** supercoiled pBR322 plasmid remained untreated or 5 ng/µL plasmid DNA incubated with 0.5 MBq [^67^Ga]GaCl_3_ **(C)**.

**Supplementary Tables**

|  | 1.25 ng/µL | | 5 ng/µL | |
| --- | --- | --- | --- | --- |
|  | $\boldsymbol{k}_{\boldsymbol{sr}}^{\boldsymbol{D}}$ **(Gy^-1^)** | $\boldsymbol{k}_{\boldsymbol{rl}}^{\boldsymbol{D}}$ **(Gy^-1^)** | $\boldsymbol{k}_{\boldsymbol{sr}}^{\boldsymbol{D}}$ **(Gy^-1^)** | $\boldsymbol{k}_{\boldsymbol{rl}}^{\boldsymbol{D}}$ **(Gy^-1^)** |
| EBRT | 1.21 ± 0.04 | 0.017 ± 0.001 | 0.45 ± 0.06 | 0.04 ± 0.002 |

Table S1. Conversion rates from supercoiled to relaxed DNA (k_sr_) and from relaxed to linear DNA (k_rl_) for plasmid DNA irradiated by EBRT at a plasmid concentration of 1.25 or 5 ng/µL. Rates summarised in this table are from Figures 2 and 4.

|  | 1.25 ng/µL | |
| --- | --- | --- |
|  | $\boldsymbol{k}_{\boldsymbol{sr}}^{\boldsymbol{D}}$ **(Gy^-1^)** | $\boldsymbol{k}_{\boldsymbol{rl}}^{\boldsymbol{D}}$ **(Gy^-1^)** |
| [^67^Ga]GaCl_3_  0.5 MBq | 0.585 ± 0.1 | 0.0025 ± 0.0009 |
| [^111^In]InCl_3_  0.5 MBq | 0.131 ± 0.01 | 0.0010 ± 0.0002 |
| [^67^Ga]GaCl_3_  1 MBq | 0.65 ± 0.4 | 0.003 ± 0.002 |
| [^111^In]InCl_3_  1 MBq | 0.271 ± 0.007 | 0.0003 ± 1e-05 |

Table S2. Conversion rates from supercoiled to relaxed DNA (k_sr_) and from relaxed to linear DNA (k_rl_) for plasmid DNA incubated with 0.5 ot 1 MBq [^67^Ga]GaCl_3_ or [^111^In]InCl_3_ at a plasmid concentraion of 1.25 ng/µL. Rates summarised in this table are from Figures 2, 3B and 4 .

|  | 5 ng/µL | |
| --- | --- | --- |
|  | $\boldsymbol{k}_{\boldsymbol{sr}}^{\boldsymbol{D}}$ **(Gy^-1^)** | $\boldsymbol{k}_{\boldsymbol{rl}}^{\boldsymbol{D}}$ **(Gy^-1^)** |
| [^67^Ga]GaCl_3_  0.5 MBq | 0.159 ± 0.009 | 0.0051 ± 0.0005 |

Table S3. Conversion rates from supercoiled to relaxed DNA (k_sr_) and from relaxed to linear DNA (k_rl_) for plasmid DNA incubated with 0.5 [^67^Ga]GaCl_3_ at a plasmid concentraion of 5 ng/µL. Rates summarised in this table are from Figures 2, 3B and 4.
